# Mapping South African allied health primary care clinical guideline activity: establishing a stakeholder reference sample

**DOI:** 10.1186/s12961-016-0145-9

**Published:** 2016-10-10

**Authors:** Janine Margarita Dizon, Karen Grimmer, Shingai Machingaidze, Pam McLaren, Quinette Louw

**Affiliations:** 1 Centre for Evidence-Based Health Care (CEBHC), Faculty of Medicine and Health Sciences Stellenbosch University, Francie van Zijl Drive, Tygerberg, 7505 Cape Town, South Africa; 2 Center for Health Research and Movement Science, University of Santo Tomas, Espana, Manila, 1018 Philippines; 3 International Centre for Allied Health Evidence (iCAHE), University of South Australia, City East Campus, P4-18 North Terrace, Adelaide, 5000 Australia; 4 Department of Physiotherapy, Faculty of Medicine and Health Sciences, Stellenbosch University, Francie van Zijl Drive, Tygerberg, 7505 Cape Town, South Africa; 5 Cochrane South Africa, South African Medical Research Council, Francie van Zijl Drive, Parow Valley, 7505 Cape Town, South Africa; 6 Disability Action Research Team (DART), Howick, KwaZulu-Natal South Africa

**Keywords:** Allied health, Clinical practice guidelines, South Africa, Primary health care

## Abstract

**Background:**

Little is known about allied health (AH) clinical practice guideline (CPG) activity in South Africa, and particularly in relation to primary health care (PHC). This paper reports on a scoping study undertaken to establish a reference framework, from which a comprehensive maximum variation sample could be selected. This was required to underpin robust sampling for a qualitative study aimed at understanding South African primary care AH therapy CPG activities. This paper builds on findings from the South African Guidelines Evaluation (Project SAGE) Flagship grant.

**Methods:**

South African government websites were searched for structures of departments and portfolios, and available CPGs. Professional AH association websites were searched for CPGs, purposively-identified key informants were interviewed, and CPGs previously identified for priority South African primary care conditions were critiqued for AH therapy involvement.

**Results:**

Key informants described potentially complex relationships between players who may be engaged in South African AH CPGs, in both public and private sectors. There were disability/rehabilitation portfolios at national and provincial governments, but no uniformity in provincial government organisation of, or support for, PHC AH services. There were no AH primary care therapy CPGs on government websites, although there was ‘clinical guidance’ in various forms on professional association websites. Only two CPGs of priority South African PHC conditions included mention of any AH therapy (physiotherapy for adult asthma and chronic obstructive pulmonary disease).

**Conclusion:**

A comprehensive and wide-reaching stakeholder reference framework would be required in order to capture the heterogeneity of AH primary care CPG activity in South Africa. This should involve the voices of national and purposively-selected provincial governments, academic institutions, consultants, public sector managers and clinicians, private practitioners, professional associations, and private sector insurers. Provincial governments should be selected to reflect heterogeneity in local economics, population demographics and availability of university AH training programs. This investigation should aim to determine the areas of PHC in which AH are engaged.

## Background

Primary health care (PHC) in South Africa is the framework by which effective, efficient, integrated and accessible care could be provided to the greatest number of people, with the greatest gains for the country [1, 2]. Primary care is the manner in which individuals enter the PHC system [3]. Primary care in South Africa was defined by Dookie and Singh [4] as services provided by general practitioners, nurses or other allied health professionals and is regarded as the first point of entry to the health system. Primary care is oriented towards health promotion and disease prevention and focuses on individuals and communities. This level of care allows for early diagnosis, management, and referral to secondary and tertiary care, thereby providing the potential for continuity of care [4].

Allied health (AH) has a significant role to play in the management of sequelae of disease, particularly when it influences function [5]. AH is an umbrella term, encompassing a diverse group of professionals who are not medical or nursing [6]. Generally, AH is considered in two streams with different tasks – therapies and diagnostic/scientific. The National Society of Allied Health, citing a 2012 consensus statement by the International Council of Health Professionals Organisation, notes that AH is “*a distinct group of health professionals who apply their expertise to diagnose, treat and rehabilitate people of all ages and all specialties. Together with a range of technical and support staff they may deliver direct patient care, health promotion, rehabilitation, treatment, diagnostics and health improvement interventions to restore and maintain optimal physical, sensory, psychological, cognitive and social functions*” [7].

The health priorities in South Africa for the last decade have largely been dominated by HIV and tuberculosis, with a significant proportion of the health budget being dedicated to these diseases [8]. Other major issues of concern that are being tackled with high priority in line with the millennium development goals are the high mortality of women and young children and their mothers, the rising threat of non-communicable and mental diseases, and the heavy burden of disease from injury and violence [9]. While these priority conditions have largely driven a focus on saving lives and survival, AH activities focus largely on morbidity rather than mortality [10]. This possibly explains the current lack of AH primary care recommendations in South African clinical practice guidelines (CPGs) assessed as part of a recent study which aimed to evaluate the quality of selected key South African CPGs available for use in primary care [11]. However, as survival rates from infectious diseases improve, so does the importance of enhancing quality of remaining life by reducing the burden of disability and improving functionality [12]. South African AH therapies have a significant role to play in managing and improving functional loss as a result of chronic managed illness (e.g. HIV, tuberculosis) or lifestyle diseases (e.g. diabetes, hypertension, and respiratory and heart disease), as well as post-trauma management (e.g. birth defects, neurological diseases and orthopaedic complaints) [13]. Given their focus on optimising function and quality of life, evidence-based engagement of AH in effective primary care activities, as well as their recognition within the health community as key in primary care, is essential for the generations to come and to ensure future optimal functionality and capacity of South African communities.

To achieve a better understanding of the AH activities relating to primary care and CPGs in South Africa, there was a need to engage all relevant AH stakeholders across multiple levels in government (national, provincial and district), as well as in the private sector. As a starting point, we decided to engage the AH therapy stakeholders. Several challenges were encountered, including the lack of a central register for CPGs for primary care in South Africa making it difficult to identify relevant CPGs (past or current) [11, 14], an understaffed unit within National PHC directorate encompassing AH, rehabilitation and disability issues, variations in how AH activities are perceived and managed at provincial level (e.g. AH engagement and referral practices in planning patient discharge from hospital to community), and the incomplete and fragmented nature of statistics on South African chronic lifestyle diseases, disability and morbidity [13]. This paper outlines the steps taken to establish a valid reference population, which was required to underpin a robust maximum variation sampling approach for a qualitative descriptive study that mapped South African AH therapy primary care CPG activities. Our research was undertaken as part of Project SAGE (South African Guidelines Excellence) [14], a South African Medical Research Council Flagship Project that investigated broad primary care CPG development or any CPG activity, implementation and uptake in South Africa. This innovative research partnership aims to improve the quality and reach of South African primary care CPGs using stakeholder-driven processes to assist effective South African CPG activities in developing, adapting, adopting, contextualising and implementing primary care CPGs [14].

## Methods

### Research questions

We wanted to explore the activities and contexts within which AH primary care therapy CPGs are developed, adapted or contextualised, implemented, and used in South Africa. We were interested in identifying who did what in the area of PHC AH CPGs in South Africa, how they did it and why, and how these CPGs addressed local contexts and were implemented and used.

### Framework

We intended to follow the Gold Standard COREQ (Consolidated Criteria for Reporting Qualitative Research) criteria [15] when reporting the findings of our research into activities and contexts of South African PHC AH guidelines. This paper reports preliminary scoping work to address items 10 and 11 of the COREQ criteria, namely, defining the sample and explaining how subjects were identified [15]. To do this, we followed a recently-reported methodology which addressed robust sampling from an uncertain reference population (determining key players in the occupational therapy management of children with autism) [16]. We sought to establish a framework which supported effective, efficient and comprehensive maximum variation sampling, for a proposed descriptive qualitative study to explore South African AH PHC CPG activities. There was little knowledge about an appropriate and inclusive reference population of AH stakeholders in PHC CPGs in South Africa, as we found no appropriate previously-published research in this area.

### Sampling

We decided to use a maximum variation sampling approach to address the uncertainty of the reference population, based on its purposeful sampling strategy, which aims for heterogeneity [16–18]. This approach is resource efficient, as it would enable us to interview a small number of individuals in each stakeholder group (providing AH voices in different settings and roles), who could potentially provide maximum diversity of responses and experiences with which to address our research questions. Although we proposed a purposive sampling strategy, rather than a strictly theoretical approach, we planned to determine the trustworthiness of our findings, by examining for data saturation in each stakeholder group during data capture.

### Data saturation

We predetermined that data saturation would be established if no new categories of information were discovered in at least the last two interviews analysed. Thus, while data saturation would not determine our ultimate sample size, the purposive sampling procedure allowed the inclusion of more informants in a particular stakeholder group, if needed [19, 20].

### Identifying stakeholder groups

From its collective experiences and knowledge, the Project SAGE research team identified key individuals using the following criteria: (1) they should be actively involved in AH therapy disciplines, rehabilitation, and/or disability activities in South Africa, and (2) their involvement can be in any of the following contexts (clinical practice, education, professional development, policymaking, etc.). They should have rich information to share to assist us in identifying relevant stakeholder groups. These individuals were interviewed, and asked to describe South African AH CPG activities as they understood them, in terms of the stakeholder groups which might be involved.

### Selecting participants

Once all relevant stakeholder groups were identified, we aimed to collate our sample using a consecutive snowballing approach within each stakeholder group [17]. Our recruitment plan was to initially contact key individuals in each stakeholder group, provide project information, and request an interview. These names could come from SAGE team members, from information provided on websites, or from telephoning AH professional associations, rehabilitation and disability organisations. We decided on a project-specific ‘stopping rule’ of three requests to the same person without response, before we stopped that recruitment attempt. If the initially-identified individuals agreed to participate, during the interview, they would be asked for names of others in that stakeholder group, or other relevant stakeholder groups, who could assist our enquiries. However, if key individuals refused the initial interview request, we would ask them for names of others in that stakeholder group, whom we could approach. We would thus recruit consecutively-identified individuals in each stakeholder group, and conduct interviews until we reached data saturation in each group.

### Identifying AH engagement in primary care CPGs

We revisited the 16 South African primary care CPGs which we previously collated and critically appraised for methodological quality [11]. The purpose of this was to identify whether AH therapies were mentioned as having a role in the management of South African priority primary diseases. These CPGs are reported in Table 1 of this paper for reference (reported earlier as Table Two in Machingaidze et al. [11]).

**Table 1:** South African primary care clinical guidelines reviewed by Machingaidze et al. [14]

Guideline name	Disease	Year	Allied health therapy inclusion
Clinical guidelines for the management of HIV & AIDS in adults and adolescents	Adult HIV	2010	No
Guidelines for the management of HIV in children	Child HIV	2010	No
Clinical guidelines: PMTCT (prevention of mother-to-child transmission)	PMTCT	2010	No
National tuberculosis management guidelines	Adult tuberculosis	2014	No
Guidelines for the management of tuberculosis in children	Child tuberculosis	2013	No
Malaria prevention guidelines	Malaria prevention	2011	No
Malaria treatment guidelines	Malaria treatment	2010	No
Standard treatment guidelines and essential medicines list for South Africa	Conditions encountered in primary care and chronic conditions	2008	No
Integrated management of childhood illnesses	Childhood illnesses	2002	No
Guidelines for maternity care in South Africa	Maternal	2007	No
Primary Care 101	Common symptoms and chronic conditions in adults	2013	No
Guideline for the management of acute asthma in adults: 2013 update	Adult asthma	2013	Yes
Guideline for the management of acute asthma in children: 2013 update	Child asthma	2013	No
Guideline for the management of chronic obstructive pulmonary disease – 2011 update	Chronic obstructive pulmonary disease	2011	Yes
South African hypertension guideline 2011	Hypertension	2011	No
The 2012 SEMDSA guideline for the management of type 2 diabetes (Revised)	Type II diabetes	2012	No

We then collected information from the websites of the National Department of Health and of all South African provincial Departments of Health regarding the activities of primary care portfolios relating to AH therapies. We also collected information from South African government websites on province statistics. We amassed information using the most recent (2014 mid-year) population estimates [21], percentage of South African land mass, number of people living per square kilometre, estimates of contributions to GDP [22], main economic activity, and number of traditional universities, to allow us to identify those provinces that would provide us with heterogeneity in the types of patients attending primary care. This was undertaken so that we would understand why CPGs might be developed or adapted for specific provincial needs.

### Study rigour

This preliminary research was undertaken to ensure the necessary attention to a rigorous sampling framework from a previously unreported set of stakeholders who were involved in, and knowledgeable about, AH PHC activities in South Africa. Without a comprehensive sampling frame in this complex area of service administration and delivery, we ran the risk of not identifying or interviewing appropriate participants, and/or not hearing from relevant stakeholder groups. This would compromise the external generalisability of the findings and their acceptability within South African settings (in identifying important issues in developing and implementing clinical practice guidelines), as well as the transferability of the findings.

The next stage of the study (reported in another publication) heard the voices of as many individuals as possible, involved in each stakeholder group, to ensure that we had explored all possible avenues for information. This paper discussed internal rigour methods such as data saturation, credibility by member checking and triangulation.

## Results and discussion

### Key informant interviews

To ensure the comprehensiveness of the sampling framework reported in this paper, we interviewed five key individuals who had a national breadth of knowledge regarding AH CPG activities, AH training and AH service delivery contexts in South Africa.

### Stakeholder groups

**Fig. 1:**
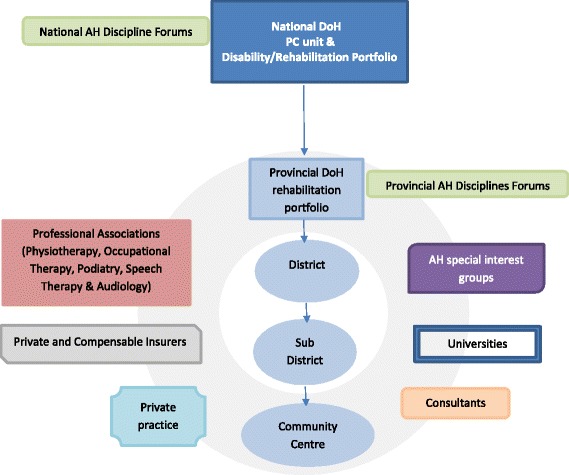
Allied health (AH) participant sampling frame. The figure outlines the areas of allied health activity which informed the sampling frame for this study. These are the areas from which participants for this study would be sought. *DoH* Department of Health, *PC* Primary Care

We identified multiple stakeholders, reflecting public and private health sectors, insurers and academia, whose voices needed to be heard to comprehensively map South African primary care AH CPG activity. Key public health stakeholders were government policymakers (National and Provincial Departments of Health in the rehabilitation/disability portfolios), South African provincial public sector administrators, hospital and clinic managers, and AH clinicians working in district and sub-district facilities. The views of participants in discipline-specific forums which assisted the rehabilitation portfolios in some provincial governments also needed to be heard. These reflected representatives of professional associations, academics, senior managers and senior clinicians. Key private sector stakeholders were professional associations, private clinicians, consultants and health insurers (Fig. 1).

This section provides more detail on the stakeholder groups.

AH discipline stakeholders, or private and publically-funded primary care AH therapy in South Africa is mostly delivered by physiotherapy, occupational therapy, podiatry and speech pathology (which includes audiology) [23]. Nutrition, which is usually considered an AH discipline, appears to stand alone from the other therapies, with its own governmental secretariats/portfolios at national and provincial levels. Thus, it was not considered further in this investigation. Physiotherapy, Occupational Therapy, and Speech and Language Therapy and Hearing are taught in almost if not all of the 10 traditional South African universities, found in seven of the nine provinces. Podiatry is currently only taught in one technical university. Orthotics/prosthetics were not included in the AH disciplines for this research, as the focus of this discipline group was on manufacture following referral, not therapy.

AH therapy is also delivered in the private sector, mostly in community-based private practices, and funded by individuals, with or without private or third party insurance. Clinicians in the private sector appear to be generally engaged in CPG activities through their professional associations or as part of higher research degrees or continuing education activities. There were also special interest or action groups such as Rural Rehabilitation South Africa. This was an informal grouping of South African AH clinicians, managers, educators and researchers with an interest in clinical guidelines and evidence translation, across sectors. These (often informal) groups were to be found at provincial or national levels, and might be multi-disciplinary, single discipline and/or condition-specific.

Medical aid (health insurance companies) had an interest in CPG activities, particularly as funders of CPG activities by professional associations, or implementers. It seemed that there were also key private consultants who provided technical writing services to professional associations, medical aids and government, whose knowledge about AH CPG activities was important.

CPG activities may also occur in AH discipline departments in South African universities. The engagement of academics may be in research, in supervision of student projects on CPGs, engagement with discipline-specific forums advising government rehabilitation portfolios or in working with professional associations, special interest groups, and private insurers on guideline issues.

There were four AH therapies (physiotherapy, occupational therapy, podiatry, and speech therapy and audiology), and at least six key stakeholder groups (provincial AH forums, AH special interest groups, universities, consultants, private practice, and private and compensable insurers) which should be included in the reference population, to ensure effective and efficient maximum variation sampling, for an investigation of South African AH primary care CPG activities. These groups are outlined in Fig. 1.

### Describing the sample

It was possible that some interviewees from some stakeholder groups may have worn more than one ‘hat’. They could be primarily a public sector clinician, who also had a role in a professional society, and also served on a forum advising government. Thus, participating individuals would need to be described in terms of their representation of all relevant stakeholder groups in order to understand whose ‘voices’ they presented.

### Presence of AH in priority condition CPGs

Physiotherapy was the only AH profession mentioned in the CPGs previously identified by Machingaidze et al. [11] for selected priority in South African primary care conditions. Physiotherapy was mentioned for adult asthma and chronic obstructive pulmonary disease [24, 25]. For adult asthma, the possibility for undesired bronchospasms after physiotherapy was noted [24], whilst for chronic obstructive pulmonary disease, physiotherapy was recommended for effective sputum production and pulmonary rehabilitation [25].

### Identifying heterogeneous provincial governments

National and Provincial Departments of Health primary care and Rehabilitation/Disability portfolios were key stakeholders. However, the policy, administrative and clinical structures differed between provinces, particularly relevant to primary care and disability portfolios. It seemed that each province focused differently on rehabilitation and discipline-specific AH activities, specific to its constituents’ needs, workforce availability and local resource constraints. To capture representative provincial voices regarding AH CPG activity, we needed to identify provinces that reflected the heterogeneity of South African demographics, economics, contribution to South African economy, and access to traditional universities. These factors would impact on AH primary care service provision in public and private sectors.

**Fig. 2:**
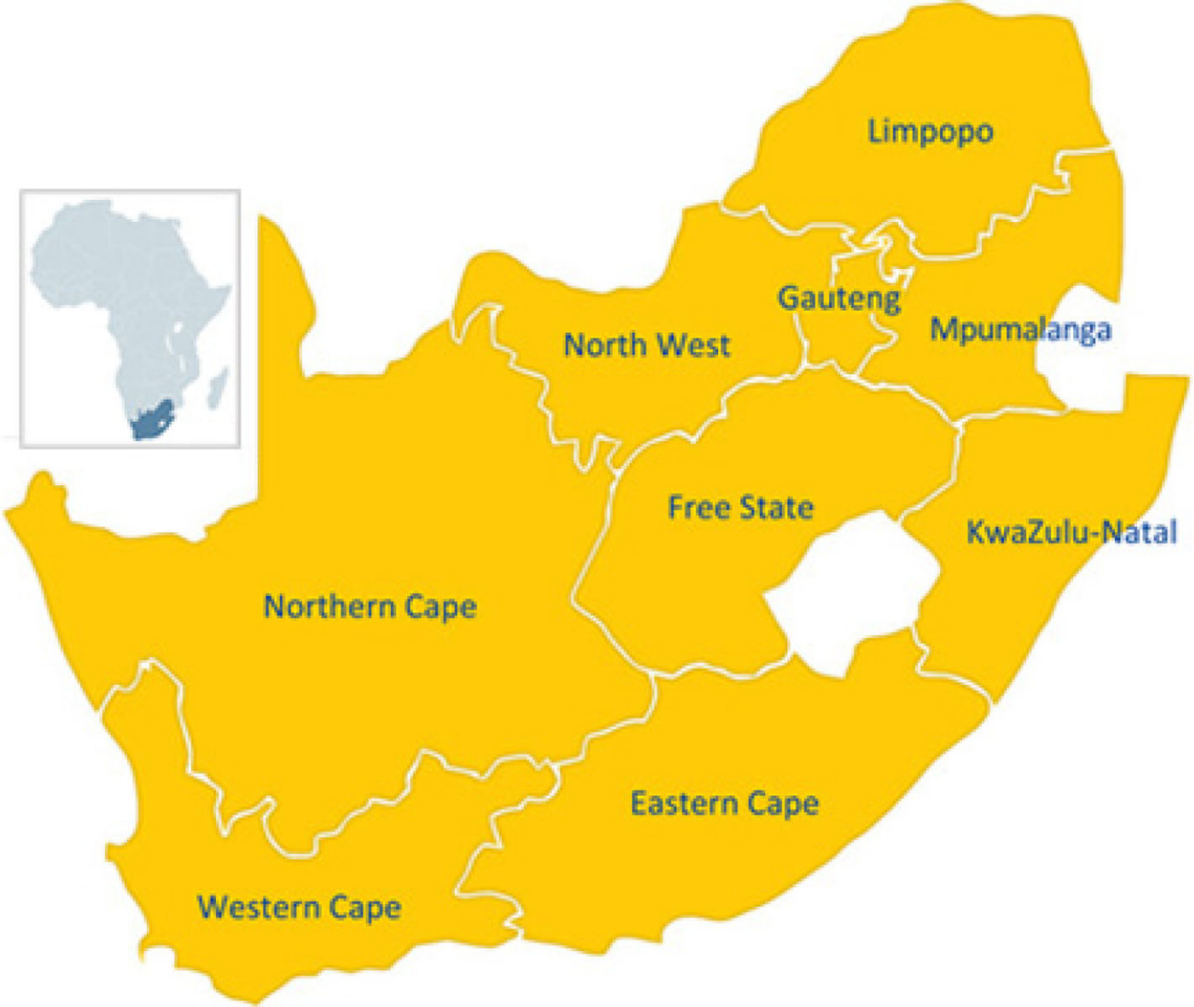
Map of South Africa

Figure 2 provides a map of South African provinces, and Table 2 provides an overview of population numbers and percent contribution to national population, percent of GDP produced, percent of land mass of South Africa, people per square kilometre and number of traditional universities per province. All provinces reported a mix of agriculture, manufacturing, mining and tourism activities; however, agriculture was the main activity in KwaZulu-Natal, Limpopo, Mpumalanga, Free State and Northern Cape, whilst manufacturing/mining was reported more in Gauteng, Eastern Cape and North West. The Western Cape reflected a mix of mining, manufacture and agriculture.

**Table 2:** South African province information

Province	Population	Percentage of National population	Percentage of GDP per capita ($US)	Share of South African area	Population density, people/km^2^	Number of traditional universities
Gauteng	12,914,800	23.9%	9681	1.4%	675	2
KwaZulu-Natal	10,694,400	19.8%	4767	7.7%	109	1
Eastern Cape	6,786,900	12.6%	3651	13.9%	39	1
Western Cape	6,116,300	11.3%	8694	10.6%	45	3
Limpopo	5,630.500	10.4%	4259	10.3%	43	1
Mpumalanga	4,229,300	7.6%	6251	6.3%	53	0
North West	3,676,300	6.8%	6677	8.7%	34	1
Free State	2,786,800	5.2%	6213	10.6%	21	1
Northern Cape	1,166,700	2.2%	6688	30.5%	3	0

After considering differences in provincial descriptors (Table 2), the provinces of Mpumalanga, Western Cape, Gauteng and Kwazulu-Natal were chosen to maximise sampling heterogeneity. Whilst Mpumalanga, Gauteng and Kwazulu-Natal are geographically close in the north-eastern aspect of South Africa, they were quite different in terms of area, population density, economy, infrastructure and tertiary education opportunities, which will facilitate a more heterogeneous and comprehensive capture of our intended sample. The Western Cape Province is situated in the south of the country, and provided a more mixed economic view, a lower population density than Gauteng and Kwazulu-Natal (but higher than Mpumalanga), and significant infrastructure and greater tertiary education opportunities than the other provinces.

## Conclusion

This paper outlines the process of establishing a defensible maximum variation sampling frame, which would underpin a future comprehensive qualitative descriptive study of AH primary care CPG activity in South Africa. We took this approach to address the suspected heterogeneity in an undefined reference population. Our reference population needed to include multiple stakeholder groups, comprising National and four heterogeneous Provincial Governments, as well as public, private, professional, business and academic sectors. By aiming for saturation of information obtained from each stakeholder group, this sampling frame should provide us with a comprehensive understanding of who, how and why AH CPGs are developed, contextualised, implemented and used in South African primary care.

## Abbreviations

AH: Allied Health
COREQ: Consolidated Criteria for Reporting Qualitative Research
CPG: Clinical Practice Guidelines
PHC: Primary Health Care
SAGE: South African Guidelines Excellence
